# Resveratrol against *Echinococcus* sp.: Discrepancies between In Vitro and In Vivo Responses

**DOI:** 10.3390/tropicalmed8100460

**Published:** 2023-09-26

**Authors:** Julia A. Loos, Micaela Franco, Maia Chop, Christian Rodriguez Rodrigues, Andrea C. Cumino

**Affiliations:** 1 Instituto de Investigaciones en Producción, Sanidad y Ambiente (IIPROSAM), Facultad de Ciencias Exactas y Naturales, Universidad Nacional de Mar del Plata (UNMdP), Funes 3350, Nivel Cero, Mar del Plata 7600, Argentina; julialoos@mdp.edu.ar; 2 Consejo Nacional de Investigaciones Científicas y Técnicas (CONICET), Mar del Plata 7600, Argentina; maiachop@mdp.edu.ar (M.C.); crodriguez@mdp.edu.ar (C.R.R.); 3 Hospital Interzonal General de Agudos “Dr. Oscar E Alende”, Mar del Plata 7600, Argentina; mfranco@mdp.edu.ar; 4 Departamento de Química, Facultad de Ciencias Exactas y Naturales, Universidad Nacional de Mar del Plata (UNMdP), Funes 3350, Nivel 2, Mar del Plata 7600, Argentina

**Keywords:** echinococcosis, resveratrol, autophagy, hematopoietic stem cell expansion, dendritic cell differentiation

## Abstract

In an attempt to find new anti-echinococcal drugs, resveratrol (Rsv) effectiveness against the larval stages of *Echinococcus granulosus* and *E. multilocularis* was evaluated. The in vitro effect of Rsv on parasites was assessed via optical and electron microscopy, RT-qPCR and immunohistochemistry. In vivo efficacy was evaluated in murine models of cystic (CE) and alveolar echinococcosis (AE). The impact of infection and drug treatment on the mouse bone marrow hematopoietic stem cell (HSC) population and its differentiation into dendritic cells (BMDCs) was investigated via flow cytometry and RT-qPCR. In vitro treatment with Rsv reduced *E. granulosus* metacestode and protoscolex viability in a concentration-dependent manner, caused ultrastructural damage, increased autophagy gene transcription, and raised Eg-Atg8 expression while suppressing Eg-TOR. However, the intraperitoneal administration of Rsv was not only ineffective, but also promoted parasite development in mice with CE and AE. In the early infection model of AE treated with Rsv, an expansion of HSCs was observed followed by their differentiation towards BMCDs. The latter showed an anti-inflammatory phenotype and reduced LPS-stimulated activation compared to control BMDCs. We suggest that Rsv ineffectiveness could have been caused by the low intracystic concentration achieved in vivo and the drug’s hormetic effect, with opposite anti-parasitic and immunomodulatory responses in different doses.

## 1. Introduction

Echinococcosis is a severe neglected chronic disease caused by tapeworms of the genus *Echinococcus* at the metacestode stage. The two most clinically relevant variants of this disease are cystic echinococcosis (CE), caused by the *Echinococcus granulosus sensu lato* (*sl*) species complex and distributed worldwide, and alveolar echinococcosis (AE), caused by *Echinococcus multilocularis* and restricted to the Northern Hemisphere [1,2]. Although the *E. granulosus* metacestode is a delineated unilocular cyst, and the *E. multilocularis* metacestode a multivesicular and infiltrative structure, both contain an outer acellular laminated layer, composed of a carbohydrate network, and an inner germinal layer, constituted of different cell types and capable of producing protoscoleces [3,4]. 

To date, the only two drugs approved for their chemotherapeutical treatment are albendazole (ABZ) and mebendazole. However, both are parasitostatic drugs that must be administered at high doses for prolonged periods and have serious adverse effects [2]. Therefore, new chemotherapeutic alternatives are necessary to be developed. Given that cell energy is a commodity that these organisms cannot directly obtain from the host, and that their energy metabolism pathways differ from those of the host, the repurposing of drugs that affect parasite energy metabolism would be a rational approach for parasite treatment. In both species, the germinal layer supplies the high metabolic demand of energy and intermediate metabolites, involving cytosolic fermentation into lactate and mitochondrial fermentation via malate dismutation [5,6]. In particular, germinal cells convert most glucose into lactate through aerobic glycolysis (the Warburg effect), facilitating the uptake and incorporation of nutrients [5,6]. It has been reported that these parasites present plasticity in mitochondrial respiration and are capable of adapting their energy metabolism to oxygen availability [7,8], while under microaerobic and anaerobic conditions, *Echinococcus* can obtain ATP through fumarate respiration, involving mitochondrial complexes I and II, and under aerobic conditions these parasites carry this out through oxidative phosphorylation, which is dependent on complexes I to V. Complex I, in the first case, and complexes II, III and IV, in the second, function as proton pumps to generate a proton gradient that drives ATP synthesis via complex V [7,8].

Recent studies have shown that buparvaquone inhibited *E. multilocularis* mitochondrial complex III and that it was effective against in vitro cultured metacestodes; however, its oral administration failed to reduce the parasite mass in a secondary mouse infection model [9]. Otherwise, the oral administration of atovaquone significantly reduced the development of the primary alveolar metacestode in the mouse liver [7], and also showed a synergic effect when co-administered with ABZ [10]. Furthermore, it was recently reported that the simultaneous inhibition of complex III using ELQ-400 (an endochin-like quinolone) and complex I using quinazoline can greatly improve anti-helminthic activity against *E. multilocularis* metacestodes via the inhibition of aerobic and anaerobic energy metabolism, respectively [11]. Additionally, Enkai et al. [8] demonstrated that ascofuranone and its derivatives inhibited both complexes II and III of the mitochondrial electron transport chain in *E. multilocularis*, showing a strong anti-parasitic effect on protoscoleces under both anaerobic and aerobic conditions. 

Previously, we have demonstrated that metformin (Met), an anti-diabetic drug with high safety for normoglycemic individuals, restricts the development of metacestodes in murine models of cystic and alveolar echinococcosis [12,13,14]. The drug acts by inhibiting mitochondrial complex I of *E. granulosus* and *E. multilocularis*, leading to the activation of AMPK, suppression of TORC1 and induction of autophagy [15,16,17]. In addition, a combination of Met with low doses of ABZ improves the antiparasitic therapy efficiency in late stages of metacestode development [12,14]. Met treatment also increased levels of parasite-released lactate, suggesting that the parasite switched to fermentation for energy generation when complex I (of both the electron transport chain and the malate dismutation) was blocked [14,15]. Targeting the Warburg effect may thus be a good strategy to hinder these parasites or even improve Met anti-echinococcal activity. 

Resveratrol (Rsv; (3,4′,5-trihydroxy-trans-stilbene)) is a natural non-flavonoid polyphenol, classified as a phytoalexin, which exhibits a plethora of pharmacological properties including antioxidant, anti-inflammatory, immune-modulator and anti-cancer activities [18,19]. Resveratrol could slow cancer cell growth by interfering with glycolysis and promoting oxidative phosphorylation [20]. Furthermore, the antiparasitic activity of the compound against various pathogens such as *Trichinella spiralis*, *Hymenolepis diminuta*, *Trypanosoma cruzi*, *Toxoplasma gondii* and *Schistosoma mansoni* has been confirmed [21,22,23,24,25]. 

In this study, we aimed to assess the anti-parasitic effects of Rsv against the larval stage of *E. granulosus* and *E. multilocularis* and discuss possible reasons for the discrepancy between Rsv activity in vitro and in vivo based on our own results on the immunomodulation of the host response with the drug.

## 2. Materials and Methods

### 2.1. Ethics Statement

Female CF-1 mice (8 weeks of age) were supplied by SENASA, Mar del Plata, and housed in specific pathogen-free facilities at the bioterium of the National University of Mar del Plata (UNMdP). Animal experiments were performed in strict accordance with the 2011 revised form of The Guide for the Care and Use of Laboratory Animals published by the U.S. National Institutes of Health. Experimental protocols for using mice were evaluated and approved by the Animal Experimental Committee at the Faculty of Exact and Natural Sciences, UNMdP (permit numbers RD 625-2021 and RD-80-2022).

### 2.2. Chemicals

Metformin (1,1-dimethylbiguanide hydrochloride) was obtained from Sigma-Aldrich (St. Louis, MO, USA) and Rsv from Santa Cruz Biotechnology (Dallas, TX, USA). For in vitro assays, trans-Rsv was kept as a 70 mM stock solution in dimethyl sulfoxide (DMSO). For in vivo experiments, an aqueous solution of Met and a 10% ethanol PBS solution of trans-Rsv (dissolution was achieved via continuous sonication at 40 W for 2 h at 25 °C in darkness) were prepared every 2 days from a solid drug and maintained under refrigeration (3–5 °C).

### 2.3. In Vitro Experiments

#### 2.3.1. Parasite Maintenance and Isolation

*E. granulosus* protoscoleces were obtained from hydatid cysts of infected cattle slaughtered in the industrial abattoir Mar del Plata (official number 3879), Buenos Aires, Argentina [26], and metacestodes were harvested from the peritoneal cavities of CF-1-infected mice via standard protocols [26]. *E. multilocularis* (isolates J2012 and 8065) was maintained via passage in CF-1 mice and the isolation of protoscoleces was carried out from mice infected as previously described [27,28]. 

#### 2.3.2. Drug Treatment and Viability Assays

*E. granulosus* protoscoleces (n = 3000) were cultured in 24-well culture plates as we described in detail previously [29] in the presence of Rsv (10, 50 and 100 μM). The murine cysts (n = 10–20) were incubated in Leighton tubes under the same culture conditions as described for protoscoleces [15]. Parasites cultured with DMSO were used as controls. Protoscolex viability was determined using the methylene blue exclusion test [26], while cyst viability was evaluated via the trypan blue staining of detached germinal layers. All experiments were carried out until the viability of the control group was lower than 90% or all treated parasites were dead. In all culture systems, the medium was changed every third day, including the fresh addition of Rsv. All experiments were carried out at least three times independently.

#### 2.3.3. Ultrastructural Analysis via Scanning Electron Microscopy

Control and treated *E. granulosus* metacestodes (10 and 50 μM Rsv for 4 days) and *E. multilocularis* parasite material recovered from each in vivo experimental group (control, 50 mg kg^−1^ day^−1^ Met, 100 mg kg^−1^ day^−1^ Rsv and 50 mg kg^−1^ day^−1^ Met plus 100 mg kg^−1^ day^−1^ Rsv) were processed for scanning electron microscopy (SEM) observation with a JEOL JSM-6460LV electron microscope as previously described [26]. 

#### 2.3.4. In Toto Immunohistochemistry

For in toto immunohistochemistry, control and pharmacologically treated metacestodes (25 μM Rsv for 48 h) were processed for the analysis of Eg-Atg8 and phosphorylated (Ser^3122^) Eg-TOR as previously described [13,17]. Twenty immunofluorescence images per condition of 3 independent sets of experiments were acquired and analyzed through protocols established in the laboratory [13]. Ratios of Eg-Atg8 or Eg-TOR-P to the fluorescence intensity of nuclei were calculated and displayed as bar plots. 

#### 2.3.5. In Vitro Culture of Bone Marrow-Derived Dendritic Cells from *E. multilocularis* Infected Mice

Untreated and drug-treated mice were sacrificed via cervical dislocation. Bone marrow-derived dendritic cells (BMDCs) were obtained by flushing the bone marrow of tibias and femurs. Cells were cultured as previously described [30]. At day 5, a pulse of 50 ng mL^−1^ Flt3L, a growth factor for the differentiation of BMDCs, was added. Lipopolysaccharide (LPS or Gram-negative bacterial endotoxin) was used in an amount of 100 ng mL^−1^ as a potent immune inducer.

#### 2.3.6. Flow Cytometry

BMDCs were harvested from culture plates, centrifuged to eliminate the medium and then suspended in solutions containing allophycocyanin (APC), phycoerythrin (PE), fluorescein isothiocyanate (FITC) or phycoerythrin cyanine 5 (PECy5)-conjugated mAbs for 15 min at 4 °C. Cells were stained with mAb directed to MHC class II (PE), CD80 (APC), CD86 (FITC), CD11c (PECy5), FLT3 (PE), Ly6G (PE), SCA-1 (FITC) and SIRPα (PE) (all from eBioscience). To ensure no bound antibodies were eliminated, BMDCs were washed with a PBS −1% FBS solution. The results were obtained using a BD FACS Canto TM II flow cytometer and are expressed as the mean fluorescence intensity or as the percentage of positive cells.

#### 2.3.7. Gene Expression Analysis via Reverse Transcription Quantitative Polymerase Chain Reaction (RT-qPCR)

Extractions of total RNA from *E. granulosus* metacestodes and BMDCs were carried out as described [26]. Reverse transcriptions were carried out using 5 μg of total RNA for metacestodes and 1–500 ng of total RNA for BMDCs in the presence of M-MLV RT (Invitrogen). The amplification of Eg-atg8 and Eg-atg12 from *E. granulosus* metacestodes was performed using previously designed primers [29]. The primers designed for Eg-tfeb were Eg-tfeb-fw (5′-ATGAATCCAACGGCCACTCATAACAG-3′) and Eg-tfeb-rev (5′-GATTAATGAGGCGTAGGTAGACTGAAC-3′), the amplicon size of which was 327 bp. *Echinococcus granulosus* ezrin-like protein (elp, GenBank accession number CBH50747) was used as a reference gene in qPCR assays [16,31].

Bone marrow dendritic cells were collected from culture after 7 days. The medium was eliminated via centrifugation and 100 μL of RNA later^®^ solution (Ambion) was added. For gene expression analysis, specific primer pairs were designed: TGF-β (fw: 5′-TTGCTTCAGCTCCACAGAGA-3′; rev: 5′-TGGTTGTAGAGGGCAAGGAC-3′), IL-10 (fw: 5′-CCAAGCCTTATCGGAAATGA-3′; rev: 5′-TTTTCACAGGGGAGAAATCG-3′), TNF-α (fw: 5′-AGCCCCCAGTCTGTATCCTT-3′; rev: 5′-CTCCCTTTGCAGAACTCAGG-3′), and IL-6 (fw: 5′-AGTTGCCTTCTTGGGACTGA-3′; rev: 5′-TCCACGATTTCCCAGAGAAC-3′). GAPDH was used as an endogenous control.

qPCR reactions were conducted under the following conditions: 94 °C for 10 min, followed by 35 cycles of 94 °C (15 s), 55 °C (30 s), and 72 °C (30 s). The qPCR product was detected using SYBR^®^Green PCR Master Mix (Applied Biosystems, Waltham, MA, USA). The relative quantification of mRNA expression was calculated in accordance with the 2−^ΔΔCt^ method. Three independent experiments were carried out, each one in triplicate and using sterile ddH2O as negative controls.

### 2.4. In Vivo Assays

#### 2.4.1. Experimental Animals and Determination of the Efficacy of In Vivo Treatment

Healthy CF-1 mice (30 ± 5 g) were acclimatized for one week before the initiation of the experiments. Mice were infected via the i.p. injection of 1000 *E. granulosus* protoscoleces in 0.5 mL of phosphate-balanced solution at pH 7.0 or 200 μL of homogenized *E. multilocularis* metacestode material (strain 8065) to produce experimental secondary cystic or alveolar echinococcosis, respectively [12,13]. The animals were maintained under controlled laboratory conditions as previously described [15]. Pharmacological treatments were performed via the intragastric or i.p. administration of drug solutions (0.2–0.35 mL per animal). At the end of the experiments, mice were anesthetized with ketamine–xylazine (50–5 mg kg^−1^ per mouse) and euthanized via cervical dislocation. At the stage of necropsy, the peritoneal cavity was opened, and the parasite tissues were carefully recovered and weighted. The efficacy of treatment was calculated using the following formula: 100 × {(mean parasite weight of control group) − (mean parasite weight of treated group)}/(mean parasite weight of control group). 

#### 2.4.2. Therapeutic Effectiveness of Resveratrol in Experimental Models of Echinococcosis

In order to evaluate the effect of Rsv on murine experimental models of echinococcosis, two different experimental designs were performed.

In an experimental model of CE, at the time of infection with *E. granulosus* protoscoleces, 20 CF-1 mice were allocated into 2 experimental groups (10 animals/group), the untreated control group (10% ethanol/PBS) and the Rsv-treated group (100 mg kg^−1^ day^−1^). The drug was daily administered by an i.p. injection for 14 days. Four months after infection, animals were euthanized and necropsied.

In an experimental model of AE, at the time of infection with *E. multilocularis* metacestode material, 40 CF-1 mice were allocated into 4 experimental groups (10 animals/group), the untreated control group (vehicle), the Rsv-treated group (100 mg kg^−1^ day^−1^), the Met-treated group (50 mg kg^−1^ day^−1^), and the Rsv (100 mg kg^−1^ day^−1^) plus Met (50 mg kg^−1^ day^−1^)-treated group. For 60 days, Rsv was administered every other day via an i.p. injection and Met daily per oral gavage. At the end of the treatment period, animals were euthanized, necropsy was carried out immediately thereafter, and intracystic Rsv concentration was determined from the cyst fluid as described below.

#### 2.4.3. Determination of Resveratrol Levels

Brilliant Cresyl Blue (BCB, phenoxazin-5-ium, 1,3-diamino-7-(diethylamino)-8-methyl-chloride, Suipacha Chemist) was used to estimate intracystic Rsv concentrations, based on the spectrophotometric quantification (Shimatzu-UV-100) of a violet chromogen (λmax of 634 nm) obtained as an oxidation product in the presence of the drug in an alkaline medium (37 mM NaClO solution, [32]). Standard curves were carried out with different concentrations of pure Rsv solutions in the range of 0.1 to 100 µM. After vortexing and incubation for 5 min in the dark at room temperature, absorbance at 634 nm was evaluated.

### 2.5. Statistics

Statistical analysis was performed using R software (version 4.2.1). Student’s *t*-test or analysis of variance (ANOVA) was used to analyze the data. When significant differences (* *p* < 0.05, ** *p* < 0.01, and *** *p* < 0.001) were observed in the ANOVA table, and a Tukey post hoc test was used to identify differences between conditions.

## 3. Results

### 3.1. Resveratrol Exerts Anti-Echinococcal Activity In Vitro by Inhibiting TOR in E. granulosus Larval Stage

**Figure 1:**
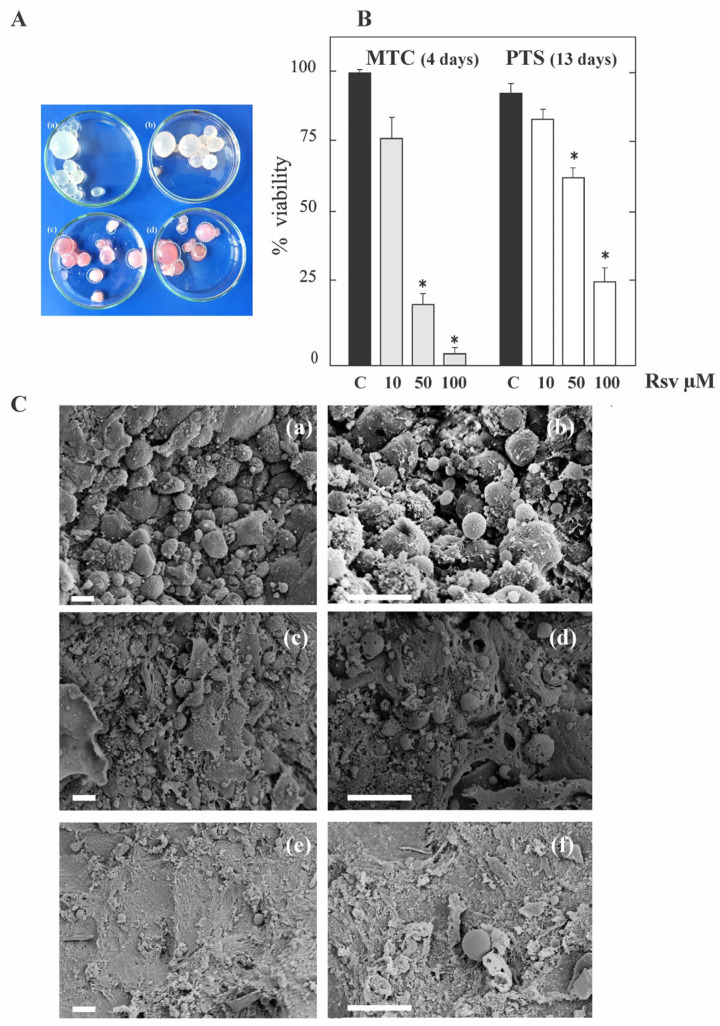
In vitro effect of resveratrol on the viability and ultrastructural characteristics of protoscoleces and metacestodes of *E. granulosus*. (**A**) Macroscopical damage of metacestodes treated with 10 μM (**b**), 50 μM (**c**) and 100 μM (**d**) resveratrol for 4 days. Control metacestodes (**a**) without morphological changes and treated metacestodes showing increased permeability (culture medium inside cysts) and collapsed germinal layer (circles). (**B**) Viability of metacestodes (MTC) and protoscoleces (PTS) incubated for 4 and 13 days, respectively, with 10, 50 and 100 μM of resveratrol (Rsv). Parasites incubated in culture medium containing DMSO served as controls (**C**). Values are the mean vitality (%) ± S.D. of three independent experiments. * Statistically significant difference (*p* < 0.05) compared with control. (**C**) Scanning electron microscopy of metacestodes incubated with 10 μM (**c**,**d**) and 50 μM (**e**,**f**) Rsv for 4 days. Control metacestode with an intact germinal layer (**a**,**b**); treated metacestodes with altered germinal layers (**c**–**f**). Bars indicate 10 μm.

To investigate the in vitro effect of Rsv on the viability of the *E. granulosus* larval stage, the proportion of dead metacestodes and protoscoleces was analyzed in response to various Rsv concentrations. After 4 days of incubation with 50 and 100 μM Rsv, metacestodes presented a detachment of the germinal layer in at least 80% of the cysts (Figure 1A). As shown in Figure 1B, 4 and 13 days of exposure led to a dose-dependent decrease in the viability of metacestodes and protoscoleces, respectively. At 50 and 100 μM Rsv, 80 ± 4% and 95 ± 2% of metacestodes and 35 ± 3% and 75 ± 5% of protoscoleces were dead, respectively. During the same time, at 10 μM Rsv, protoscoleces revealed no significant changes in vitality in comparison to the control and approximately 25 ± 8% of metacestodes were dead. In addition, Rsv-induced ultrastructural damage was evidenced via SEM. While control metacestodes exhibited an intact germinal layer (Figure 1(Ca,b)), those treated with Rsv displayed a marked loss of cells in them, showing a larger damaged area at 50 μM (Figure 1(Ce,f)) compared to 10 μM (Figure 1(Cc,d)).

On the other hand, the effects of Rsv on the autophagy pathway and its regulation by TOR were investigated in metacestodes incubated with the drug. Via qPCR, we found that the transcript levels of Eg-atg8, Eg-atg12 and Eg-tfeb increased two-, two-and-a-half-, and three-fold in metacestodes treated with 25 μM Rsv for 48 h, respectively, relative to those of the control (Figure S1A). Additionally, via in toto immunolocalization assays, Eg-Atg8 (a LC3β-homolog) was detected in a diffuse and punctate form in both control (Figure S1(Ba,b)) and 25 μM Rsv-treated metacestodes (Figure S1(Bc,d)), with the total fluorescence signal and the amount of punctuated structures being higher in the presence of the drug. As shown in Figure S1C, 25 μM Rsv treatment resulted in the inhibition of Eg-TOR, as demonstrated by a decrease in the phosphorylation of Eg-TOR (Ser^3122^) in treated metacestodes (Figure S1(Cc,d)) compared with that of untreated metacestodes (Figure S1(Ca,b)).

### 3.2. Resveratrol Shows Therapeutic Ineffectiveness in Early Infection Models of Cyst and Alveolar Echinococcosis

**Figure 2:**
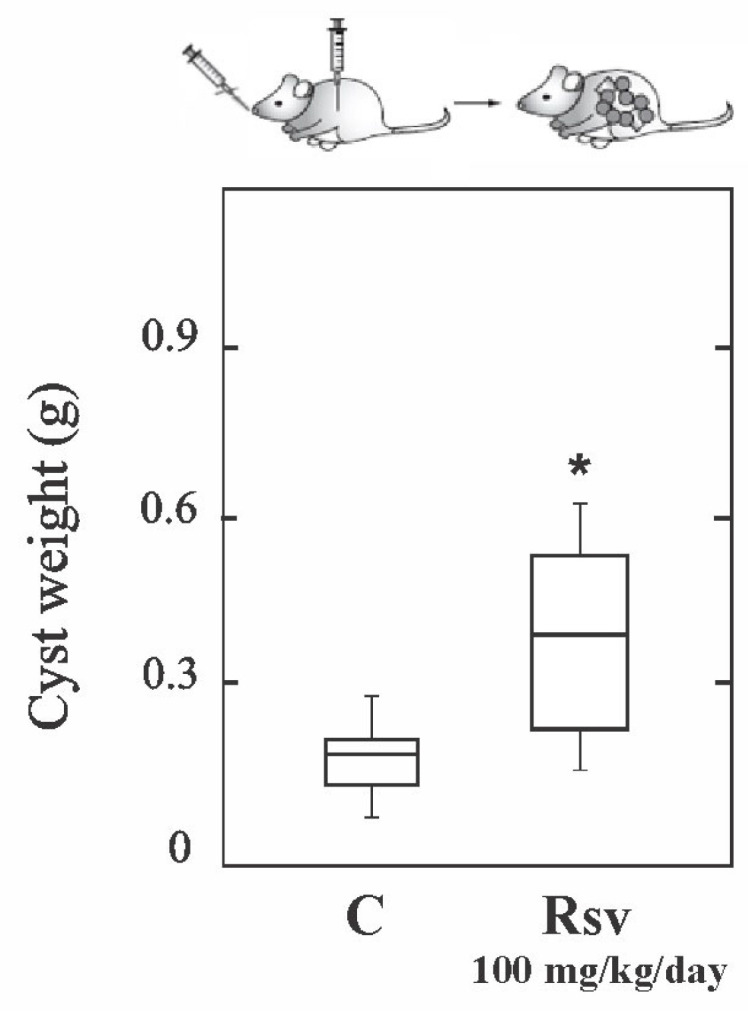
In vivo ineffectiveness of resveratrol against the *E. granulosus* larval stage. Box plot showing the comparative distribution of the weight (in grams) of cysts recovered from untreated (C) and resveratrol treated (Rsv, 100 mg kg^−1^ day^−1^) mice. A significant cyst weight increase (*, *p* < 0.05) was observed in treated animals.

Based on our in vitro results, we examined whether or not Rsv could affect parasite growth in vivo. To perform this, CF1 mice were intraperitoneally injected with protoscoleces and treated from the day of infection via the i.p. administration of the vehicle or Rsv (100 mg kg^−1^ day^−1^) over a period of 14 days. At 4 months p.i., mice were sacrificed in order to recover the hydatid cysts from their abdominal cavities. Contrary to expectation, the weight of cysts obtained from Rsv-treated mice (0.48 ± 0.20 g) was significantly higher (*p* < 0.05) than those recovered from untreated mice (0.24 ± 0.08 g, Figure 2). No adverse effects were observed in the group of treated mice.

**Figure 3:**
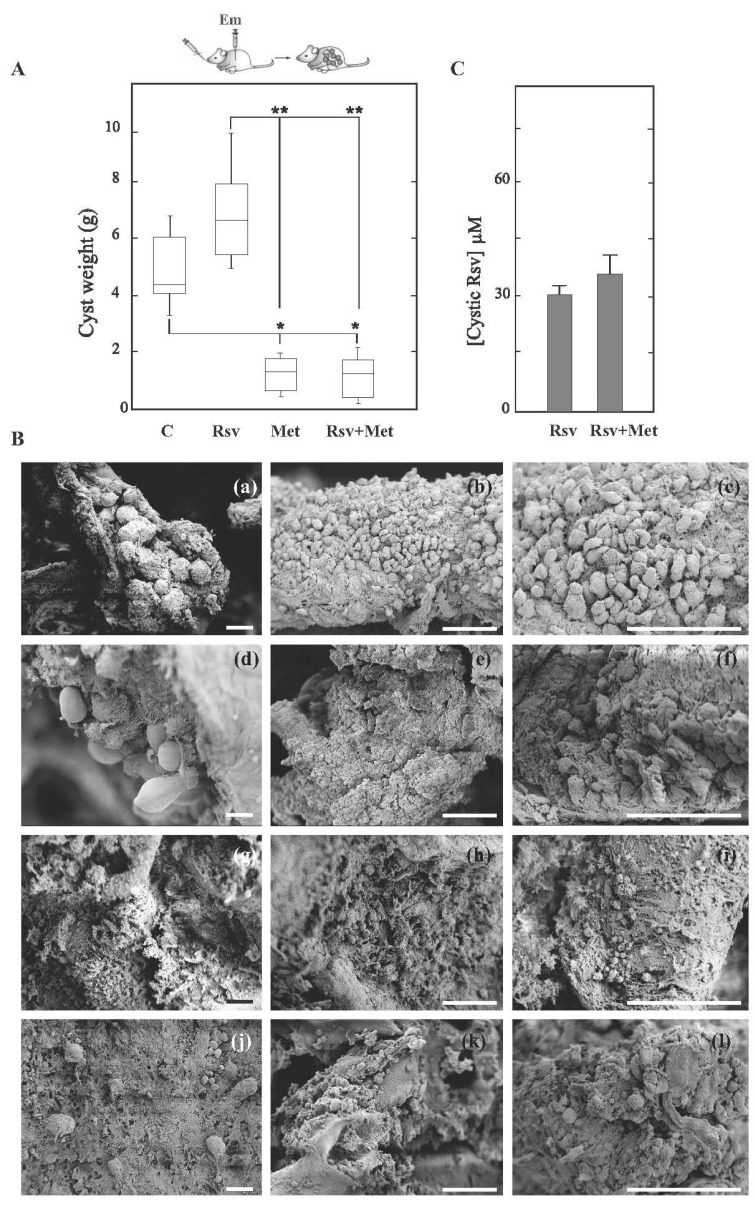
In vivo efficacy of metformin and its combination with resveratrol against the *E. multilocularis* larval stage. (**A**) Box plot showing the comparative distribution of the weight (g) of metacestodes recovered from untreated mice (C) or treated with resveratrol (Rsv, 100 mg kg^−1^ day^−1^), metformin (Met, 50 mg kg^−1^ day^−1^) and the combination of both drugs (Met + Rsv) for 60 days from the day of infection. Rsv was administered every other day via an i.p. injection and Met daily per oral gavage. A significant metacestode weight reduction (* *p* < 0.05; ** *p* < 0.01) was achieved with Met alone or combined with Rsv. (**B**) Representative SEM images of metacestodes recovered from untreated mice (**a**–**c**) or treated with Rsv (**d**–**f**), Met (**g**–**i**) and Met + Rsv (**j**–**l**). Bars indicate 50 μm (**C**) Intracystic concentration of Rsv (μM) from cysts recovered from Rsv- or Met+Rsv-treated mice in the experiment indicated in (**A**).

We have previously reported that after i.p. infection of mice with 200 μL of *E. multilocularis* metacestode material (8065 strain), the oral administration of Met (50 mg kg^−1^ day^−1^) for 8 weeks from the day of infection produced a significant reduction in parasite weight [13]. Here, we extend the study and, after confirming the in vitro sensitivity of *E. multilocularis* protoscoleces to Rsv, we evaluate the in vivo efficacy of Rsv alone or in combination with Met in the AE early infection model. In this case, drugs were administered daily per oral gavage (Met) or every other day via an i.p. injection (Rsv) from the day of infection and over a period of 60 days. All mice survived until the end of the experiment and showed no signs of stress. As shown in Figure 3A, both Met alone and combined with Rsv led to a significant reduction (*p* < 0.05) in parasite weight (1.7 ± 1.2 g and 1.7 ± 1.3 g, respectively) compared to that of the untreated (5.2 ± 2.6 g) or Rsv-treated (7.0 ± 2.8 g) groups. It should be added that Rsv treatment tended to increase parasite weight compared to that of the untreated group.

To analyze the ultrastructural changes in parasite material recovered from each experimental group, SEM studies were performed. Metacestode tissue from control mice appeared with protoscoleces and an intact germinal layer (Figure 3(Ba–c)). Likewise, cysts from Rsv-treated mice exhibited a germinal layer with a large number of cells and protoscoleces protruding from it (Figure 3(Bd–f)). In contrast, metacestodes collected from mice treated with Met (Figure 3(Bg–i)) or the drug combination (Figure 3(Bj–l)) displayed germinal layers with a loss of cells.

Furthermore, Rsv concentration was measured in cysts obtained from Rsv- or Rsv-plus-Met-treated mice using liquid from the cysts of untreated mice as a negative control (Figure 3C). The drug concentration was 30 ± 2 μM in samples from Rsv-treated animals and 35 ± 5 μM in those receiving both drugs.

### 3.3. E. multilocularis Induces the Expansion of Hematopoietic Stem Cells in the Bone Marrow of Mice

**Figure 4:**
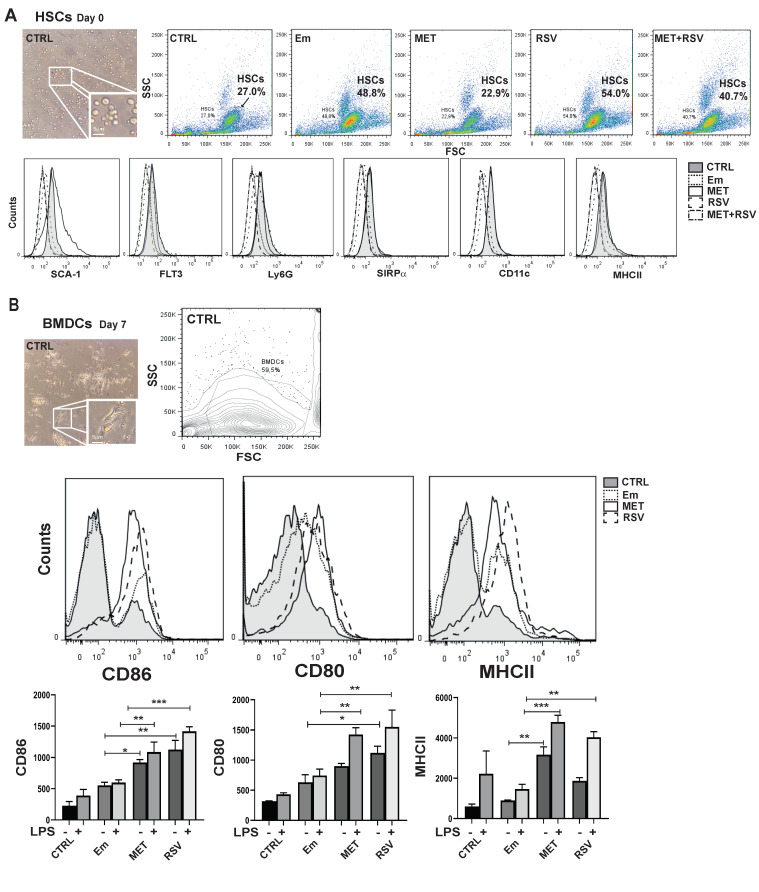
(**A**) Accumulation of bone marrow hematopoietic progenitors from alveolar echinococcosis mice coincides with parasite expansion. (**Top**) Morphological characterization of control HSCs via optical microscopy at day 0 of bone marrow isolation after 60 days post-infection. Representative dot plot graph of bone marrow HSCs gated through FSC/SSC. Numbers within dot plots represent mean HSC percentage in total bone marrow. (**Bottom**) Relative mean fluorescence intensity of Sca-1, Flt3, Ly6G, SIRPα, CD11c and MHCII for BMDCs cultured for 7 days in vitro. CTRL (HSCs from uninfected mice); Em (HSCs from AE-infected mice); MET (bone marrow HSCs from metformin-treated infected mice); and RSV (bone marrow HSCs from resveratrol-treated infected mice). (**B**) Phenotype of BMDCs in the steady state based on the expression of co-stimulatory and MHCII molecules. (**Top**) Optical photograph of BMDCs cultured in vivo from drug-treated mice. Histograms illustrating the phenotype of BMDCs in the steady state based on the expression of CD86, CD80 and MHCII in the gate of CD11c+cells for BMDCs. Grey-filled histograms represent controls. (**Bottom**) Bar graph showing the relative mean fluorescence intensity (MFI) of CD86, CD80 and MHC class II of three independent experiments in the gate of CD11c+ cells for BMDC cultures from unstimulated cells (−) or with 100 ng mL^−1^ of LPS (+). Results are the mean ± SD of 3 experiments analyzed via a one-way ANOVA test, followed by a Tukey post hoc test. *** *p* < 0.001; ** *p* < 0.01; * *p* < 0.05. CTRL (BMDCs from uninfected mice); Em (BMDCs from AE-infected mice); MET (BMDCs from metformin-treated infected mice) and RSV (BMDCs from resveratrol-treated infected mice).

Hereafter, we examined the possibility that infection with *E. multilocularis* and/or treatment with Rsv and/or Met could induce modifications to cells in mouse bone marrow. Since hematopoiesis is essential for the full induction of immune responses and it is activated in response to infections, we evaluated the potential impact of the drugs on the hematopoietic stem cell population in the murine model of early AE infection. The flow cytometry analysis showed that at day 0 of the extraction, bone marrow cells expressed the markers associated with hematopoietic progenitors, and thus they represented hematopoietic stem cells (HSCs, Figure 4A). Phenotypically, HSCs expressed the stem cell antigen-1 (Sca-1+), and were negative for CD11c- Ly6G- SIRPα- and Flt3- (Figure 4A bottom) in all conditions analyzed. Likewise, in all cases the same rounded cell morphology was observed via light microscopy (Figure 4A). Although HSC phenotype remained unchanged between the analyzed conditions, via FSC/SSC plot analysis we detected that two months after infection the relative abundance of HSC-like cells was proportional to parasite burden (Figure 4A), suggesting that they increased turnover rate as a function of the systemic demand of myeloid cells. We found that the number of HSCs increased at 60 days post-infection in untreated mice (HSC 50 ± 3%), Rsv-treated (55 ± 5%) and Rsv+Met-treated mice (41 ± 4%) (Figure 4A bottom), relative to HSCs from uninfected- (25 ± 3%) and Met-treated mice (23%, which were similar to that of uninfected mice). However, after 7 days of culture under the previously indicated conditions, HSCs could be correctly differentiated into dendritic cells without showing significant differences in common monocyte markers (Sca-1-, and CD11c+ Ly6G+ CD172a+ CD135+; Figure S3).

### 3.4. E. multilocularis Infection and Pharmacological Treatments Promotes BMDC Maturation

Given that dendritic cells (DCs) are a unique type of professional antigen-presenting cell capable of inducing naïve T cell activation and differentiation to initiate an adaptive immune response, we investigated the impact of infection and drug treatments on mouse BMDC differentiation. In cells obtained under all conditions studied, we evaluated the acquisition of maturation markers after exposure for 7 days to Flt3-L (fms-like tyrosine kinase 3 ligand). BM cells were able to fully differentiate into BMDCs, showing a marked change in cell morphology and forward and side scatter via flow cytometry (Figure 4B top). The HSC marker Sca-1 was downregulated at the cell membrane analyzed, and differentiation dendritic cell markers were upregulated (Ly6G, MHCII, CD11c, SIRPα, and Flt3; Figure S3). Also, we evaluated how these BMDC maturation markers respond to bacterial LPS stimulation. Figure 4B shows that *E. multilocularis* infection tended to up-regulate the expression of CD86, CD80 and MHCII in comparison with that of uninfected mice and that the expression change was increased via the use of LPS. A significative up-regulation of CD80 and CD86 (*p* < 0.05) was observed in BMDCs from Rsv-treated mice compared to those of untreated infected mice, and positive modulation was statistically significant with LPS stimulation in BMDCs from Rsv- and Met-treated mice. Although MHCII expression in membranes was up-regulated in BMCDs from Met-treated mice (*p* < 0.05) and followed the same trend in cells from Rsv-treated mice, the increase was significant for both treatment conditions in the presence of LPS (*p* < 0.01). These results show that Rsv and Met treatment improve the expression of BMDC co-stimulatory molecules, leading to the induction of DC phenotypic maturation. Therefore, we can conclude that the bone marrow of mice, regardless of whether they are infected or infected and drug-treated, maintain the ability to differentiate competent BMDCs.

### 3.5. Resveratrol Modifies the Cytokine Production of BMDCs from E. multilocularis-Infected Mice

**Figure 5:**
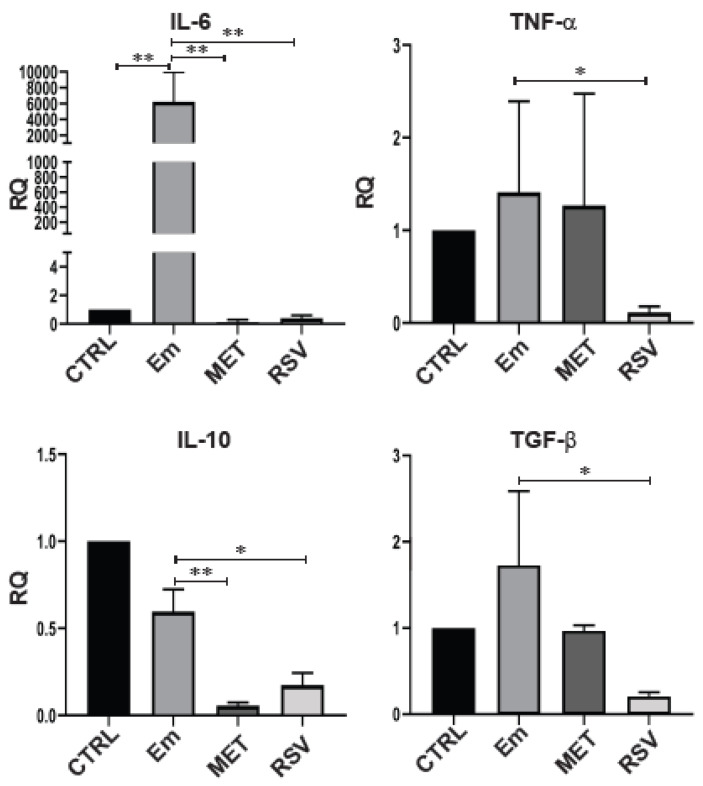
Pro- and anti-inflammatory cytokine gene expression from BMDCs obtained from AE-infected and drug-treated mice. Quantitative PCR analysis of IL-6, TNFα, IL-10 and TGFβ from total RNA of BMDCs incubated for 18 h from uninfected (CTRL), AE-infected (Em), metformin-treated infected mice (MET) and resveratrol-treated infected mice (RSV). GAPDH was used as a housekeeping gene expression. Fold change expression values (RQ) are plotted. Data are the mean RQ ± S.D. of three independent experiments. Statistically significant differences (** *p* < 0.01; * *p* < 0.05) compared with control (CTRL or Em indicated in all cases).

Finally, we analyzed and compared the mRNA levels of selected cytokines from unstimulated and LPS-stimulated BMDCs obtained from infected and treated mice. BMDCs from *E. multilocularis*-infected mice had significantly higher mRNA levels of IL-6 (*p* < 0.01) and showed a trend towards higher TGFβ expression compared to unstimulated DCs (control), while TNFα and IL-10 mRNA levels remained the same as those in the uninfected control DCs (Figure 5). The transcription of IL-6 (*p* < 0.01), TNFα (*p* < 0.05), TGFβ (*p* < 0.05) and IL-10 (*p* < 0.05) was blocked in BMDCs from Rsv-treated infected mice, whereas IL-6 (*p* < 0.01), TGFβ (*p* < 0.05) and IL-10 (*p* < 0.05) were inhibited, and TNFα was unchanged, in those from Met-treated infected mice, compared to BMDCs from untreated infected-mice. As it has been described that Rsv treatment reduces LPS-induced BMDC activation, we compared between unstimulated and LPS-stimulated BMDCs and found that treatment with Rsv and Met attenuated IL-6 and TGFβ but increased IL-10 and TNFα relative to BMDCs from untreated infected mice (*p* < 0.05, Figure S2).

## 4. Discussion

Echinococcosis is one of the most serious parasitic zoonotic diseases in the world for which most patients are diagnosed at an advanced stage of the disease when therapy with benzimidazoles is limited [33,34]. Resveratrol has been reported to be an effective anti-parasitic and anti-proliferative agent in vitro without affecting normal tissues and cells, eliminating any in vivo harmful side effects [35,36]. In this study, we examined the in vitro effects of Rsv on the *Echinococcus* larval stage, and reported its in vivo effects on early murine infection models of CE and AE.

Resveratrol decreased the viability of *E. granulosus* protoscoleces and metacestodes in vitro and induced distortion and a loss of cells in the germinal layers of the cysts. Similarly, previous studies on *H. diminuta*, *Raillietina echinobothrida*, *S. mansoni* and *T. spiralis*, resulted in the death of at least 50% of parasites with Rsv concentrations ranging from 50 to 110 μM [21,22,25,37]. However, while in these helminth parasites Rsv showed in vitro toxic effects in the short term, a longer treatment was needed to achieve similar results for *E. granulosus*.

Interestingly, Rsv was found to be a powerful chemopreventive and chemoprotective drug that modulates cancer cell death pathways in vivo and in clinical trials for drug combinations and induces autophagy and the inactivation of TOR signaling in vitro [36,38,39,40,41,42,43,44]. In this context, TOR inactivation and consequent autophagy activation in Rsv-treated cysts provides molecular evidence of the effectiveness of Rsv at concentrations greater than 50 μM.

The in vivo effects of Rsv as an anti-protozoal agent have recently been demonstrated. Vilar-Pereira et al. [45] tested the i.p. effectiveness of Rsv (15 mg kg^−1^ from 60 to 90 days post-infection) in BALB/c mice infected with *T. cruzi* and showed that the drug reduces the parasite burden in established Chagas disease. Mousavi et al. [46] demonstrated that Rsv (20 and 40 mg kg^−1^ Rsv for 28 days) has anti-leishmanial effects in promastigote-infected mice. Additionally, Pais-Morales et al. [47] evaluated the amoebicidal activity of oral treatment with Rsv (30 mg −100 mg kg^−1^- every 8 h) in hamsters inoculated with *Entamoeba histolytica* trophozoites. In this case, animals treated two days before and ten days after inoculation with trophozoites did not develop or developed very small liver abscesses. Rsv has not yet been applied to clinical practice, as it can be quickly metabolized in its glucuronidated and sulfated forms and is excreted via urine within 4 h [48]. In humans, the oral absorption of Rsv is rapid (peak in plasma is at 1.5 h) and single doses from 0.5 to 5 g have been reported to achieve maximum plasma concentrations from 73 to 539 ng mL^−1^ (0.3–2.4 μM), respectively [48]. This way, the i.p. administration of Rsv has been considered to be suitable for the treatment of abdominal malignancies such as colorectal, liver, gastric and ovarian cancers in animal models [49]. Based on this, we applied the drug through the i.p. route to increase the probability of achieving plasma levels with anti-echinococcal activity in mice. However, and contrary to expectations, the i.p. administration of Rsv was not effective in neither the murine model of CE nor that of AE. This could be because the intracystic concentrations of Rsv achieved in the in vivo assays (30 ± 2 μM) were lower than those considered effective in our in vitro experiments. Although our analytical measurement method could have detected cis- and trans-Rsv in the samples, we suggest that it must be only have detected trans-Rsv, given that drug stability would have been maintained when delivered via the i.p. route, as has been described in other mesenteric systems [49,50]. One possible explanation for these results could be the poor solubility and bioavailability of Rsv [51], and consequently reduced drug entry into parasitic tissue. In fact, Rsv precipitates were found on the abdominal mucosa of drug-treated mice. An advantage of using Met as an anti-echinococcal drug is that it reaches effective concentrations in the cyst [12]. Thus, regardless of the pharmacological effect of Rsv, its combination with Met did not prevent Met from achieving the control of parasite development, this being due to its high stability and bioavailability, as well as the ability to penetrate the cysts, conducive to achieving an effective concentration of Met in the parasite [12,13,14]. In addition, a SEM study revealed that, although germinal layers of metacestodes obtained from Rsv-treated mice exhibited some kind of damage, they showed evident parasitic development with the presence of protoscoleces. In contrast, germinal layers of metacestodes collected from mice receiving Met monotherapy and combination treatment exhibited overall cell loss compared with those from control mice, which showed an intact germinal layer filled with different cell types.

On one hand, we consider that Rsv ineffectiveness could be due to the fact that high doses of this drug may be necessary to achieve therapeutic efficacy [52]. In cancer models, it has been previously reported that low concentrations of Rsv (0–44 μM) promoted cellular growth and migration, while high concentrations of the drug (88–350 μM) inhibited cell proliferation, inducing autophagy and finally apoptosis [40]. Therefore, the increase in parasite development observed in our in vivo experiments could have been a consequence of the proliferative effects exerted by Rsv at low doses [40], which may have been counteracted by Met in the drug combination, probably due to the impact of both drugs on the same signal transduction pathways [12,17]. 

Additionally, Rsv provokes the modulation of immune functions, strengthening the immune system at low doses, and weakening it at high doses, possibly leading to immunosuppression [53]. Given that *Echinococcus* metacestode growth is clearly correlated with host immune processes [54], we analyzed the number and phenotype of HSCs at day 0 of bone marrow extraction and their differentiation to BMCDs after 1 week of in vitro culture. It has previously been described that chronic infections lead to HSC expansion with the exhaustion and dysfunction of this cell progeny [55,56,57]. Our results showed that *E. multilocularis* infection induced HSC expansion within 60 days post-infection, when parasite development was evident in the abdominal cavity and/or liver, without changes during subsequent myeloid maturation toward BMDCs. In fact, during early infection, the degree of expansion of HSCs was proportional to the weight of the parasite masses recovered from each condition of the in vivo assay. Interestingly, the population size of HSCs recovered from Met-treated mice was similar to that from uninfected mice. In the same line of evidence, it has been reported that HSC population expands and positively correlates with an increase in parasite load during protozoal and helminth infections (such as multiple *Leishmania* species, *T. gondii*, *Litomosomoides sigmodontis*, *T. spiralis*, *Heligmosomoides polygyrus*, *Trichuris trichiura* and *Fasciola hepatica).* Although in some cases the multipotent progenitor cells were modified, they also expanded in splenic and hepatic monocytic cells capable of differentiating into granulocytes, macrophages, and DCs [57,58,59,60]. Here, BMDCs from AE-infected and drug-treated mice showed an increase in surface molecules after 7 days of differentiation, demonstrating their maturation (CD80 and CD86) and activation (MHCII), compared with those of BMDCs from uninfected mice, realizing their subsequent capacity to activate T cells. Other receptors on the surface of BMDCs that mediate their activation are Toll-like receptors (TLR) such as as TLR4, which is activated in the presence of LPS yielding highly stimulated DCs in the AE mouse model [61,62]. Under LPS stimulation, all analyzed BMDCs from treated infected mice evidenced an up-regulation of co-stimulatory molecules with respect to those under the same conditions without LPS. Thus, BMDCs from infected AE mice preserved their functional capacity for presenting antigens.

Based on the extensive characterization of the anti-AE adaptive immune response [63,64,65], we analyzed the functional state of dendritic cells in control and drug-treated mice, as they are critical antigen-presenting cells implicated in the activation of previously characterized naïve T cells in mice with secondary AE [61,62] and a key therapeutic target in promoting the potential anti-inflammatory activity of Rsv [66,67]. We showed that BMDCs from an early stage of AE infection expressed high levels of IL-6 and TGF-β, while IL-10 and TNF-α gene expression levels were persistently low in concordance with previous reports [68,69]. As a standard positive control, LPS-stimulated BMDCs expressed high levels of IL-6 and TNFα, two cytokines that promote inflammation, but also up-regulated IL10 and showed low levels of TGF-β, both mediators of anti-inflammatory activity. Interestingly, IL-6 and TGF-β secreted by DCs facilitate transcriptional differentiation programs in naïve T cells, which is essential for the generation of Th17 cells, depending on the expression of the transcription factors RORγt and RORα [70]. In contrast, under LPS stimulation, BMDCs from Rsv-treated mice showed reduced IL-6, TNF-α and TGF- β mRNA, as well as increased IL-10, in line with the anti-inflammatory effects described for Rsv in other in vitro and in vivo cell models [52,53,66,71]. These cytokine changes probably occur given that Rsv-targeted signaling pathways are associated with a decrease in the activation of the transcriptional factors STAT3 and NF-κB, as has been described in different cell lines, including DCs [53,72]. The cytokine assays showed that Rsv significantly reduced the production of pro-inflammatory mediators as IL-6 and TNF-α and increased the expression of anti-inflammatory mediators as IL-10 in LPS-induced DCs. This mechanism could be attributed mainly to the suppression of the NFkB and MAPK signaling cascades via the inhibition of the TLR4 signaling pathway [73,74,75]. Surprisingly, Rsv and derived drugs repressed Th17 differentiation and promoted Treg maturation [76,77], which is precisely the opposite effect to that which resolves *E. multilocularis* infection, through a Th1/Th17 increase and Treg reduction [64,78].

Resveratrol has been characterized as a hormetic compound as it exhibits a dose-dependent biphasic effect [79] and is capable of inducing opposite biological effects in different doses. Therefore, it will be critical to identify effective and specific doses for this dual-responsive drug as a potential anti-proliferative and/or immunopotentiator agent for the treatment of echinococcosis. In conclusion, Rsv shows anti-echinococcal activity in vitro by targeting TOR kinase, a relevant developmental signaling hub in *Echinococcus* cells. However, these results could not be reproduced in vivo, probably because of its poor absorption and unfavorable pharmacodynamic properties. Therefore, Rsv would not be a promising substance neither for direct topical applications such as in the intestine for deworming canids nor as an drug for the percutaneous aspiration–injection–reaspiration (PAIR) method for cystic echinococcosis. Although an excessive concentration of Rsv damaged the germinal layer of the parasite, the drug showed negative immunological and anti-proliferative effects in the mouse model of echinococcosis. Thus, our assays do not allow us to propose the use of Rsv as a drug for the treatment of CE or AE.

## Data Availability

All data generated or analyzed during this study are included in this published article.

## Supplementary Materials

The following supporting information can be downloaded at https://www.mdpi.com/article/10.3390/tropicalmed8100460/s1. Figure S1: Effect of resveratrol on the expression of key autophagy genes, Eg-ATG8 and Eg-TOR-P^3122^ in metacestodes of *E. granulosus*; Figure S2: Gene expression of pro- and anti-inflammatory cytokines from BMDCs under LPS stimulation for each studied condition. Figure S3: Differentiation of BMDCs analyzed by flow cytometry from drug-treated mice. HSCs (1 × 10^6^/mL) were obtained from metformin plus resveratrol (MET+RSV) treated mice and cultured for 7 days in complete medium supplemented with Flt3-l. Optical microscopy with typical morphology and histograms illustrating the expression of Sca-1, CD11c, CD135 (Flt3), CD172a (SIRPα and Ly6G).
