# Supplementary material for: Resveratrol against Echinococcus sp.: Discrepancies between In Vitro and In Vivo Responses
Source: Trop Med Infect Dis. 2023 Sep 26;8(10):460. doi: 10.3390/tropicalmed8100460 (PMC10610609; doi:10.3390/tropicalmed8100460)
Supplement: Supplementary file 1 [file tropicalmed-08-00460-s001.zip › tropicalmed-2545198-supplementary.pdf]

Supplementary Materials

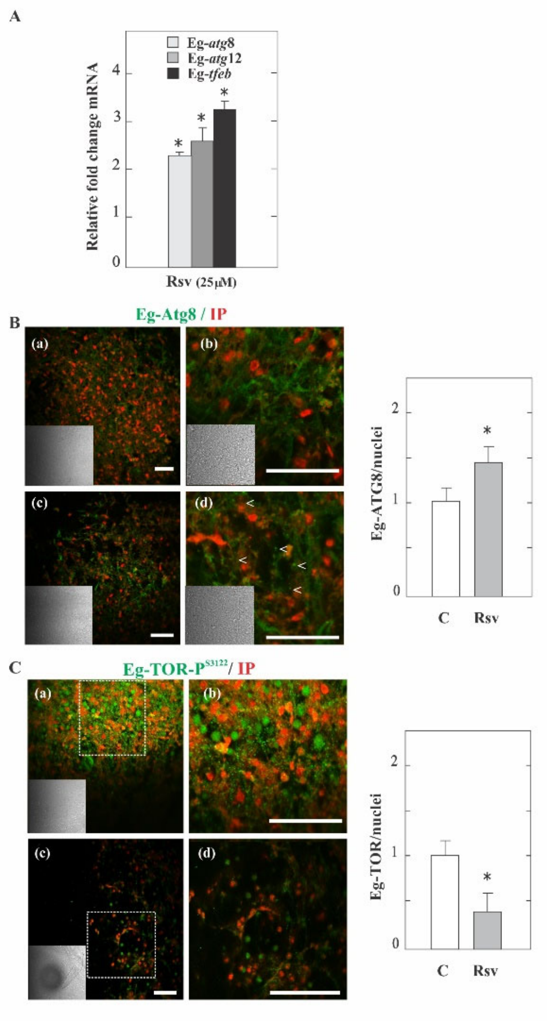

Figure S1. Effect of resveratrol on the expression of key autophagy genes, Eg-ATG8 and Eg-TOR-P3122 in metacestodes of *E. granulosus*. (A) Quantitative PCR analysis from total RNA of metacestodes treated with 25 μM resveratrol (Rsv) for 48 h compared to control. Amplification of Eg-ezrin like protein (elp) was used as a reference gene. Fold change expression values are plotted. Data are the mean ±S.D. of three independent experiments. *Statistically significant difference ($p < 0.05$) compared with control. (B) Representative confocal images of in toto immunolocalization assays revealed with an antibody conjugated with Alexa 488 (green fluorescence) and counterstained with propidium iodide (red fluorescence). Control (a,b) and Rsv-treated (c,d) metacestodes incubated with anti-LC3 antibody. Inset images correspond to transmission microscopy. Bars indicate 50 μm. Graph depicts the ratio of Eg-Atg8 to nuclei fluorescence intensity in metacestodes treated with 25 μM Rsv relative to controls. Values are expressed as means ± standard error of the mean (*, $p < 0.05$ compared to the control). (C) Representative confocal images of in toto immunolocalization assays revealed with an antibody conjugated with Alexa 488 (green fluorescence) and counterstained with propidium iodide (red fluorescence). Control (a,b) and Rsv-treated (c,d) metacestodes incubated with anti-TOR-P. Inset images correspond to transmission microscopy. Bars indicate 50 μm. Graph depicts the ratio of Eg-TOR-PS3122 to nuclei fluorescence intensity in metacestodes treated with 25 μM Rsv relative to controls (C). Values are expressed as means ± standard error or the mean (*$p < 0.05$ compared to the control).

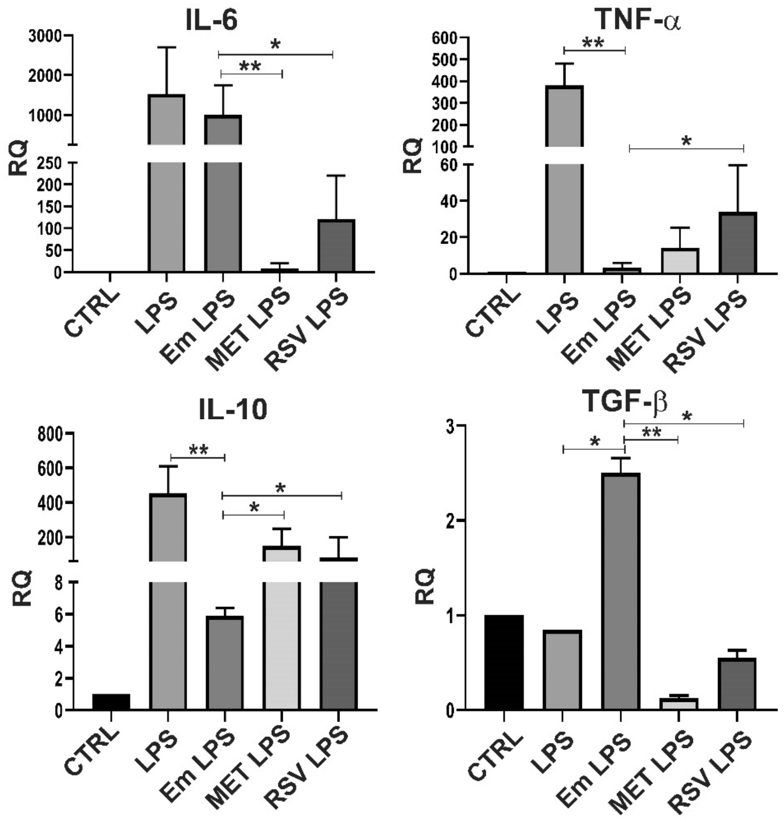

Figure S2. Gene expression of pro- and anti-inflammatory cytokines by BMDCs under LPS-stimulation for each studied condition. Quantitative PCR analysis of IL-6, TNFα, IL-10 and TGFβ from total RNA of BMDCs incubated for 18 h from uninfected (CTRL), AE infected (Em), metformin treated AE mice (MET) and resveratrol treated AE mice (RSV) in presence of 100 ng/ml LPS. Fold change expression values are plotted and GAPDH gene was used as a reference. Data are the mean ± S.D. of three independent experiments. Statistically significant differences (**p < 0.01; *p < 0.05) compared with uninfected and LPS-stimulated control cells.

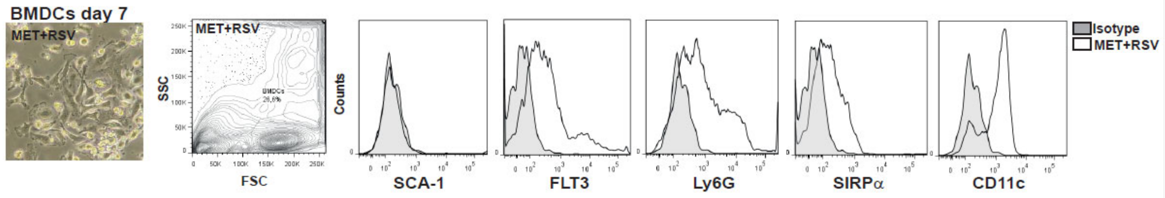

Figure S3. Differentiation of BMDCs analyzed by flow cytometry from drug-treated mice. HSCs (1 × 10^6/ml) were obtained from metformin plus resveratrol (MET+RSV) treated mice and cultured for 7 days in complete medium supplemented with Flt3-l. Optical microscopy with typical morphology and histograms illustrating the expression of Sca-1, CD11c, CD135 (Flt3), CD172a (SIRPα and Ly6G).
